# Evaluation of the Effect of the Dynamic Behavior and Topology Co-Learning of Neurons and Synapses on the Small-Sample Learning Ability of Spiking Neural Network

**DOI:** 10.3390/brainsci12020139

**Published:** 2022-01-21

**Authors:** Xu Yang, Yunlin Lei, Mengxing Wang, Jian Cai, Miao Wang, Ziyi Huan, Xialv Lin

**Affiliations:** School of Computer Science and Technology, Beijing Institute of Technology, Beijing 100081, China; 3120201035@bit.edu.cn (Y.L.); maplemon1988@163.com (M.W.); 3120201001@bit.edu.cn (J.C.); 3220201093@bit.edu.cn (M.W.); 3220200891@bit.edu.cn (Z.H.); 3220201066@bit.edu.cn (X.L.)

**Keywords:** small-sample learning, spiking neural network, structural learning, adaptive structure

## Abstract

Small sample learning ability is one of the most significant characteristics of the human brain. However, its mechanism is yet to be fully unveiled. In recent years, brain-inspired artificial intelligence has become a very hot research domain. Researchers explored brain-inspired technologies or architectures to construct neural networks that could achieve human-alike intelligence. In this work, we presented our effort at evaluation of the effect of dynamic behavior and topology co-learning of neurons and synapses on the small sample learning ability of spiking neural network. Results show that the dynamic behavior and topology co-learning mechanism of neurons and synapses presented in our work could significantly reduce the number of required samples, while maintaining a reasonable performance on the MNIST data-set, resulting in a very lightweight neural network structure.

## 1. Introduction

One of the most significant disadvantages of Deep-Leaning based Artificial Intelligence (AI) is the dependence on large amounts of tagged data samples [1,2,3]. Since small-sample learning ability or zero-shot learning ability is one of the most significant characteristics of the human brain, brain-inspired AI research has become very popular in recent years.

Researchers have explored many different brain-inspired technologies or architectures to construct a neural network that could achieve human-like intelligence. Although the human brain has not been explored exhaustively, the extraordinary ability of the human brain may be due to three basic observations: adaptive-and-flexible connectivity, structural-and-functionalized tissue levels, and spatial-and-timing dependent spiking communication [4].

The study of brain-inspired AI has given birth to the upsurge of research on Spiking Neural Networks (SNN). As the third generation of ANN [2], SNN is much more similar to the human brain. It uses spiking neurons as a computing unit, uses synapses to connect neurons to form a complex neural network, and uses spiking signals to exchange and transmit information. A neuron unit in the SNN is active only when it receives or fires a spiking signal, so it is event-driven and adopts an asynchronous working mode, which can significantly save energy consumption. And the spiking signals used to exchange information in the SNN are inherently time-dimensional and can record valuable information more sparsely [1].

In this work, we present our effort at constructing a brain-inspired SNN. We choose the LIF (Leaky-Integrate-and-Fire) model [5] to build our SNN, after comparison with other spiking neuron models [6,7].

The contributions of our work are:

- Implemented a structural learning process based on Hebbian Rule [8,9] and Use-and-Disuse theory [10] to achieve adaptive-and-flexible connectivity in our neural network;
- Implemented a reinforcement learning-based supervised synaptic learning process to achieve spatial-and-timing dependent spiking communication;
- Explored a cluster-grouped structure mode to mimic the structural-and-functionalized tissue levels of the human brain, which is inspired by the fact that neurons would usually firstly form into homogeneous clusters as the primary processing unit in the human brain’s neural network, and the working mechanism of a society where individual independent behaviour finally converges into group behaviour.

This paper is organized as follows: related works are discussed in Section 2; our method is introduced in detail in Section 3; results are presented and discussed in Section 4; and the conclusion is given in Section 5.

## 2. Related Works

Since SNN is more similar with the human brain in the aspect of biological characteristics, it has drawn a lot of researchers into this domain [11,12,13,14,15,16,17,18,19]. Many learning algorithms for SNN have already been presented.

In 2013, Beyeler et al. presented their hierarchical spiking neural network (SNN) that integrates a low-level memory encoding mechanism with a higher-level decision process to perform a visual classification task in real-time [20]. Their SNN consists of Izhikevich neurons and conductance-based synapses for realistic approximation of neuronal dynamics. And the learning method for their SNN is a spike-timing-dependent plasticity (STDP) synaptic learning rule with additional synaptic dynamics for memory encoding, and an accumulator model for memory retrieval and categorization.

In 2017, Srinivasan et al. [21] presented a neuronal potential and spike-count based enhanced learning scheme which additionally accounts for the spiking frequency to further the efficiency of synaptic learning. Also in 2017, Matsubara et al. proposed a SNN learning model which could adjust synaptic efficacy and axonal conduction delay in both unsupervised and supervised manners [22]. The same year, Iyer et al. [23] and Shrestha et al. [24] also presented their unsupervised learning algorithms for SNN.

Cho et al. presented their 2048-neuron globally asynchronous locally synchronous (GALS) spiking neural network (SNN) chip in 2019 [25]. They allow neurons to specialize to excitatory or inhibitory, and apply distance-based pruning to cut communication and memory for the sake of scalability. Rathi et al. presented a sparse SNN topology where noncritical connections are pruned to reduce the network size, and the remaining critical synapses are weight quantized to accommodate for limited conductance states [26]. In their work, pruning is based on the power law weight-dependent spike timing dependent plasticity model; synapses between pre- and post-neuron with high spike correlation are retained, whereas synapses with low correlation or uncorrelated spiking activity are pruned. Also in that year, Zhao et al. presented an SNN with an energy-efficient and low-cost processor that was based on a mechanism with increased biological plausibility, i.e., a frequency adaptive neural model instead of a Poisson-spiking neural model [27].

In 2020, Qi et al. proposed a hybrid framework combining the convolutional neural networks (CNNs) and SNNs named deep CovDenseSNN, such that SNNs can make use of the feature extraction ability of CNNs during the encoding stage, but still process features with the unsupervised learning rule of spiking neurons [28]. In 2021, Tsur proposed a method based on the gradient descent method. Their method uses the Neural Engineering Framework (NEF), which has also been evaluated with CNNs and DNNs [29].

## 3. Cluster-Grouped Structural-and-Synaptic Co-Learning Method

As discussed before, the extraordinary ability of the human brain may be due to three basic observations: adaptive-and-flexible connectivity, structural-and-functionalized tissue levels, and spatial-and-timing dependent spiking communication. We undertake the building of our SNN inspired by those three characteristics. In this paper, the MNIST [30] data-set is chosen as the evaluation data-set.

### 3.1. Overview of Our SNN Structure

**Figure 1:**
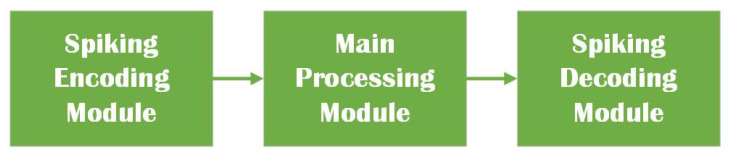
The overall structure of our SNN.

Our SNN could be roughly divided into three modules, as shown in Figure 1.

The Spiking Encoding Module is used to transfer external input into spiking signals, which are then fed to the Main Processing Module. The Spiking Decoding Module is used to comprehend and interpret the output of the Main Processing Module.

**Figure 2:**
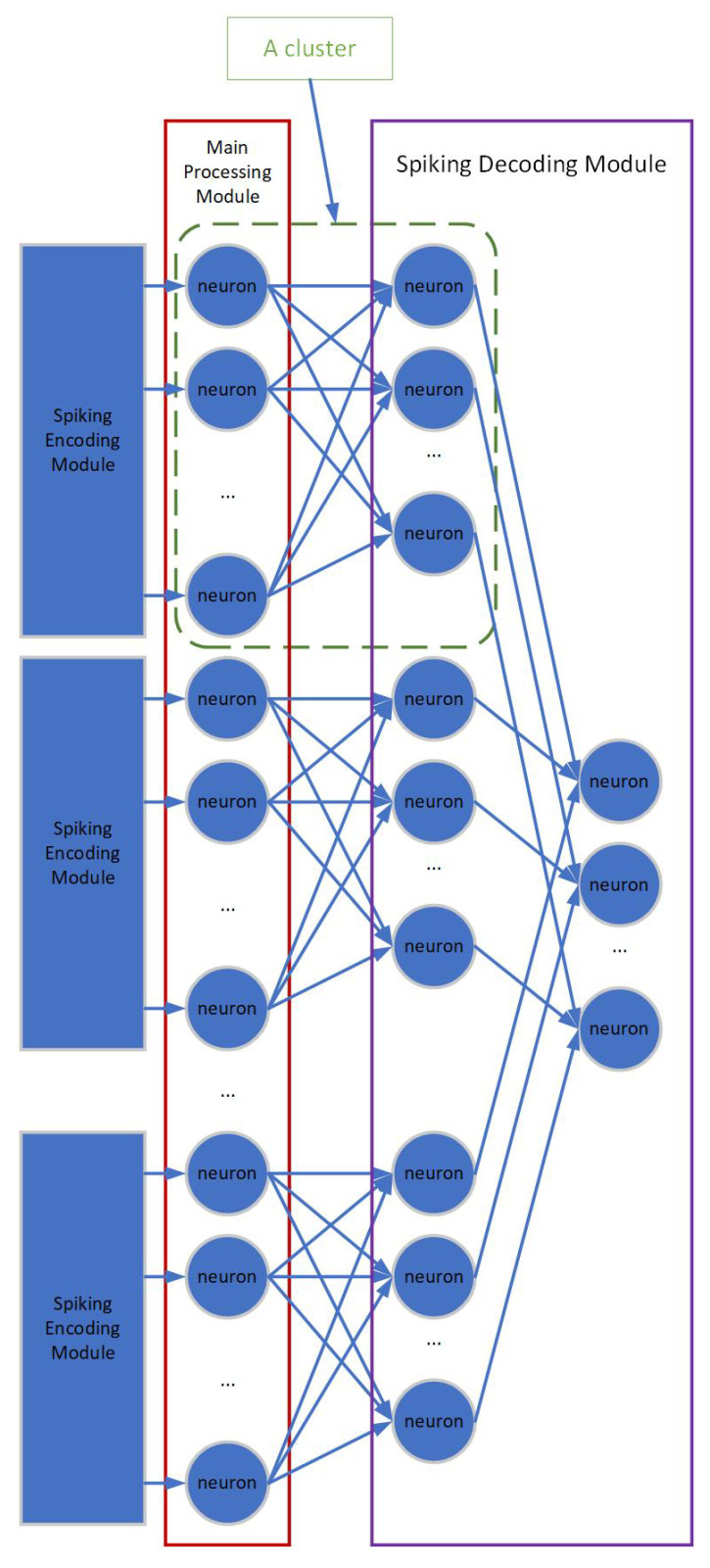
Illustration of the cluster-grouped structure mode of our SNN.

As discussed before, we explored a cluster-grouped structure mode to mimic the structural-and-functionalized tissue levels of the human brain. In the human brain, the neurons would form into many homogeneous clusters. And those clusters would serve as the basic units for the brain’s neural network. In society, group behaviour is a combination of individual behaviour. Inspired by those facts, we have grouped the Main Processing Module and the Spiking Decoding Module of our SNN into many homogeneous clusers, serving as the basic units for the proper functioning of the whole neural network, as shown in Figure 2.

#### 3.1.1. Spiking Encoding Module

The task of the Spiking Encoding Module is to encode as much useful information into spatiotemporal spike patterns as possible. Inspired by the biological visual neural system, we design our Spiking Encoding Module into two parts: feature extraction and spiking conversion.

According to biological research, the structure of the front-end feature extraction part of the visual neural network of higher organisms is relatively regular, which is mainly formed by construction rather than learning. It serves as a preliminary feature extraction phase before further feature abstraction performed by the neural network. Convolution and pooling methods are widely used in the Deep-learning Neural Networks (DNNs), and have achieved significant results.

**Figure 3:**
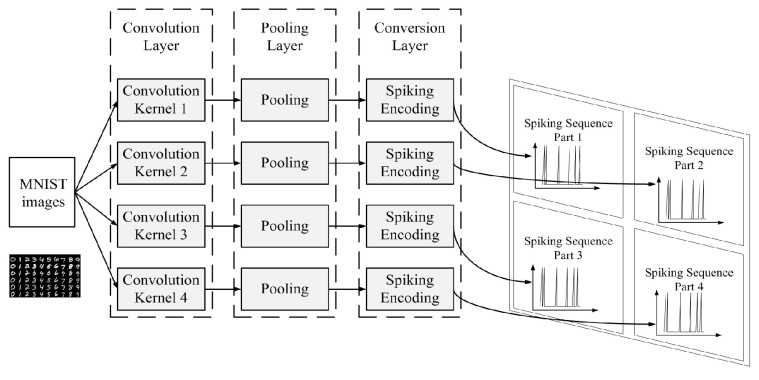
The flow of the Spiking Encoding Module [31].

In previous work, we have already designed a convolution and pooling combined preprocess method which could serve as a front-end feature extraction phase of our SNN. The flow of our Spiking Encoding Module is shown in Figure 3. The Spiking Encoding Module consists of three layers: A convolution layer, a pooling layer, and a conversion layer. The detail description of this module can be found in [31]. For the completeness of this work, we will briefly introduce it here.

Four convolution kernels are used in the convolution layer. Then, the result of the four convolution kernels would be processed by the pooling layer. Finally, the conversion layer will be used to encode the output of the pooling layer into spiking sequences [31].

The ROC (Rank Order Coding) coding method [32] is used to help design the encoding method in this paper. The spiking encoding method used in this paper converts the pixel value of the image into the delay time of the spiking signal, and the higher the pixel value is, the shorter the delay time is [31].

#### 3.1.2. Main Processing Module

The Main Processing Module consists of many homogeneous clusters. And each cluster consists of a layer of neurons. The input to the Main Processing Module is the spiking sequences generated by the Spiking Encoding Module. The number of neurons in each cluster is decided according to the number of pixels in the processed image (after convolution and pooling processing).

#### 3.1.3. Spiking Decoding Module

The task of Spiking Decoding Module is to comprehend and interpret the results generated by the Main Processing Module. The Spiking Decoding Module consists of two layers of neurons. The first layer of the Spiking Decoding Module consists of many clusters as a match to the Main Processing Module. However, the number of neurons in each cluster of the first layer of the Spiking Decoding Module is decided by the specific task requirement. And the number of neurons in the second layer of the Spiking Decoding Module is the same as the number of neurons in each cluster of the first layer of the Spiking Decoding Module.

For example, in this work, since we chose the MNIST data-set as the evaluation data-set, the number of neurons in each cluster of the first layer of Spiking Decoding Module is 10, as a correspondence to the 10 categories of MNIST data-set. Also, the number of neurons in the second layer of the Spiking Decoding Module is 10.

A full connection is adopted for the corresponding cluster in the Main Processing Module and the cluster in the first layer of the Spiking Decoding Module.

### 3.2. Structural-and-Synaptic Co-Learning Method

The flow of our structural-and-synaptic co-learning method is shown in Algorithm 1.
**Algorithm 1** Structural-and-Synaptic Co-Learning Method for Our SNN**Input:**     Training Set *D*, which consists of *n* labeled samples  Number of clusters in the SNN *T***Output:**     Trained SNN *N*1:Use Spiking Encoding Module to convert *D* into Input Spiking Sequence Set *S*;2:Initialize the SNN *N*;3:Set cluster index t=1;4:Construct training input spiking sequence sub-sets $S_{1}$, $S_{2}$,..., $S_{T}$ through bootstrap sampling;5:**while** (t≤T) **do**6:   Perform structural-and-synaptic co-learning on $N_{t}$ with $S_{t}$;7:   t=t+1;8:**end while**

We will explain the structural and synaptic learning process in detail separately.

#### 3.2.1. Synaptic Learning Process

In this paper, we presented a reinforcement learning based supervised synaptic learning process to achieve spatial-and-timing dependent spiking communication in our SNN. We rely on Spike-Timing-Dependent Plasticity (STDP) and reinforcement learning to generate our supervised learning method.

According to the STDP learning rule, the adjustment of weight of a synapse should comply with causality presented by the input spiking sequences. The STDP learning rule is used as a core learning rule in many supervised learning algorithms because of its good biological interpretability. However, lots of existing supervised learning algorithms based on the STDP learning rule only depend on the guidance of unipolar supervised signals (namely excitatory supervised signals).

According to biological researche, there are two kinds of neurons in the human brain: excitatory neurons and inhibitory neurons. It is the balance between those two kinds of neurons that supports the proper functioning of the human brain. So, in this paper, we build a bipolar supervised learning method for SNN based on STDP and reinforcement learning.

The flow of our bipolar supervised learning algorithm is described in Algorithm 2.
**Algorithm 2** Bipolar Supervised Learning Algorithm (BS)**Input:**     Input spiking sequence sub-set *S*  Un-trained cluster *N***Output:**     Trained cluster *N*1:**while** (S≠ϕ) **do**2: Pick one input spiking sequence *s* labelled with *L* from *S*;3: Feed *s* to *N*;4: Record the firing behaviour of neurons in layer 1 of Spiking Decoding Module of cluster *N*;5: Build neuron set M={x|xfired};6: **while** (*M* not only contains the neuron corresponding to label *L*) **do**7:  **for** each x∈M **do**8:   **if** *x* corresponds to label *L* **then**9:    Build encouraging teacher signal for *x*;10:   **else**11:    Build punishing teacher signal for *x*;12:   **end if**13:  **end for**14:  Feed *s* to *N*;15:  Record the firing behaviour of neurons in layer 1 of Spiking Decoding Module of cluster *N*;16:  Build neuron set M={x|xfired};17: **end while**18: Delete *s* from *S*;19:**end while**

**Figure 4:**
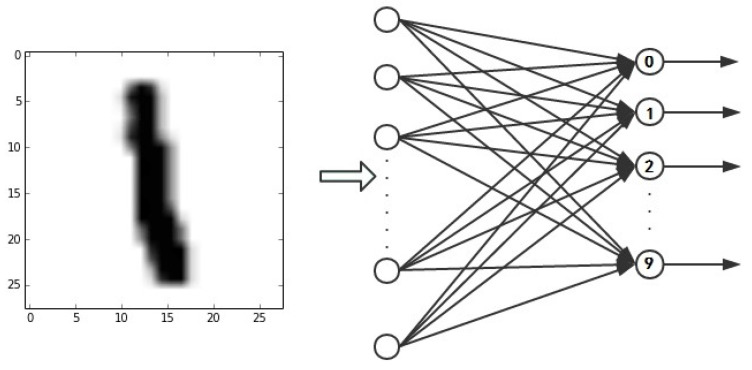
An example to illustrate bipolar supervised learning mechanism.

Suppose now we are training one of the cluster *N* shown in Figure 4.

As we can see, the image now used as the training input is an image labeled as “1”. That image would be converted into a spiking sequence first, then fed to the Main Processing Module of a cluster *N*. Let us assume during this turn of training that the set *M* of neurons fired in layer 1 of the Spiking Decoding Module of cluster *N* is [0,1,7,8], where we simply use the corresponding label of that neuron to identify it. So, we can see that the fired set of neurons not only contains the neuron that corresponds to label “1”. Thus, we need to build encouraging teacher signal for neuron corresponds to label “1”, to give it a positive reward. And build punishing teacher signals for neurons correspond to label “0”, “7”, and “8”, respectively, to give them negative reward.

#### 3.2.2. Structural Learning Process

The most significant characteristis of the biological neural network is that it could adjust its structure as a reflection to the external environment to build an adaptive neural network structure. However, lots of current research about SNNs exploit the fixed structure; the learning process of SNNs isonly focused on optimizing the weight of synapses. So the benefits of a biological neural network have not been fully explored.

The method based on fixed structure and connection is not conducive to efficient learning: On one hand, redundant connections will increase training time and lead to higher training costs; on the other hand, redundant connections will interfere with the correct results and affect the recognition accuracy. Therefore, to enhance the structural efficiency of SNN, and further leading to the energy efficiency of the SNN, we have designed a structural learning process which could dynamically and adaptively adjust the SNN structure.

There are mechanisms of synapse enhancement, weakening, and even extinction in the real biological neural network, similar to the principle of “use and disuse theory” [10] in biological evolution. In this paper, the synaptic learning process could achieve the enhancement and weakening of synapses, so the structural learning process mainly focuses on the implementation of the synapse extinction mechanism.

If the weight of some synapses remains unchanged or does not change much during the learning process, then the synapse is not involved in learning, meaning that it is redundant. Redundant synapses would be eliminated during the structural learning process in order to achieve an adaptive neural network structure.

For example, if synapse *i* has an initial weight $W_{initial}\left(i\right)$. During the structural learning process, the algorithm would check synapse *i*’s current weight, say $W_{current}\left(i\right)$. A threshold is predefined as $W_{change}$. If the following condition is satisfied, then synapse *i* would be eliminated.

(1) $$|W_{current}\left(i\right)-W_{initial}\left(i\right)|<W_{change}$$

## 4. Experiments and Results

### 4.1. Experiment Setup

**Figure 5:**
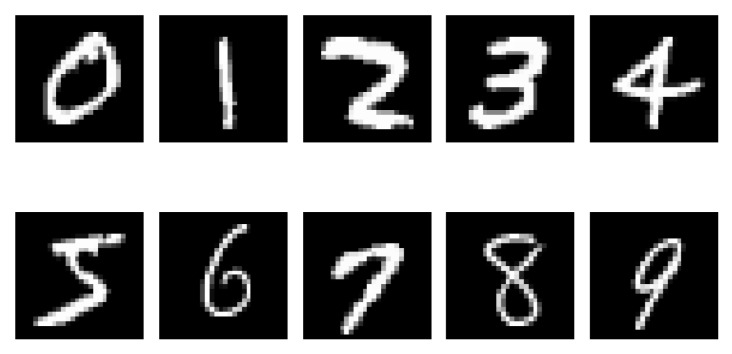
Example of MNIST data-set.

MNIST data-set [30] is chosen to test our proposed SNN. MNIST is a widely used data-set for optical character recognition with 60,000 handwritten digits in the training set and 10,000 in the testing set. The size of handwritten digital images in this data-set is 28 × 28, as shown in Figure 5.

We built our simulation platform based on the NEST (NEural Simulation Tool) [33]. NEST is a simulation platform specially designed for SNN research which promotes the exchange of computational neuroscience methods and the transfer of computer science knowledge to neuroscience. NEST follows the logic of laboratory electrophysiological experiments and provides more than 50 neuronal models and more than 10 synaptic models according to the neuron models published in related literature. The neural network in NEST consists of two basic element types: nodes and connections. NEST is suitable for SNNs of any size. It provides a method to check and modify the state of spiking neurons at any time during the simulation. NEST only takes up a small amount of memory and has high efficiency, reliability and scalability.

Biological spiking neural networks are characterized by the parallel operation of thousands of spiking neurons and the exchange of information between them by spiking trains sent by synapses. This mode of work fits the characteristics of the MPI (Message Passing Interface) parallel mechanism in particular. NEST supports MPI parallelization. NEST provides users with a method of asynchronous multi-process concurrent execution, which makes the program execute asynchronously efficiently and automatically synchronizes the process in the simulation process without user interaction. Parallel computing reduces computing time and improves the scale of operations.

### 4.2. Evaluating Convolution and Pooling Configuration

Inspired by the mechanism of biological visual information processing, we propose to use convolution and pooling to perform preliminary feature extraction. According to our study, using more than one layer of convolution or pooling does not bring enough enhancement to accuracy to compensate for the increase of network complexity and learning time, so we only use one convolution layer and one maximum_pooling layer.

**Figure 6:**
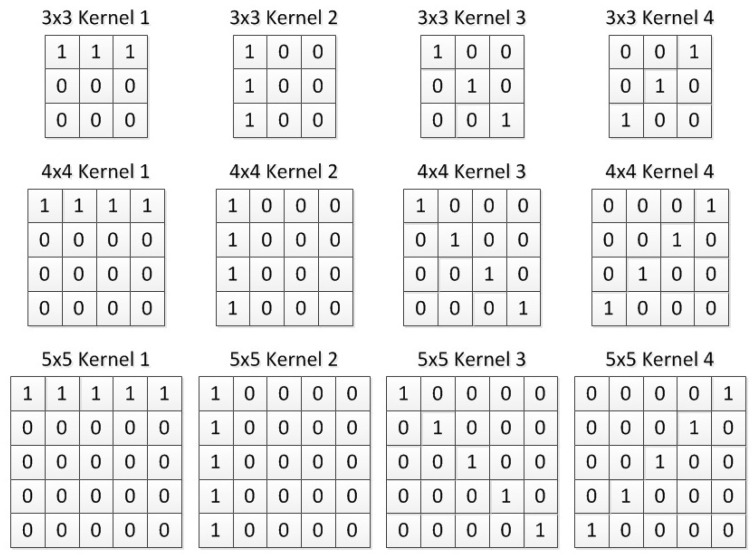
Convolution kernel of different size.

At present, the size of the convolution kernel commonly used in DNN is 3×3, 4×4, 5×5, and the pooling size is 2×2, 3×3, 4×4. So, in this work, we also choose a convolution kernel of 3×3, 4×4, 5×5, and the pooling size is 2×2, 3×3, 4×4. Our research shows that when using convolution kernels with transverse, longitudinal and two cross diagonal weights distributions, the core features of the image can be obtained more clearly. Figure 6 shows the convolution kernels of different sizes that are used in this paper.

**Figure 7:**
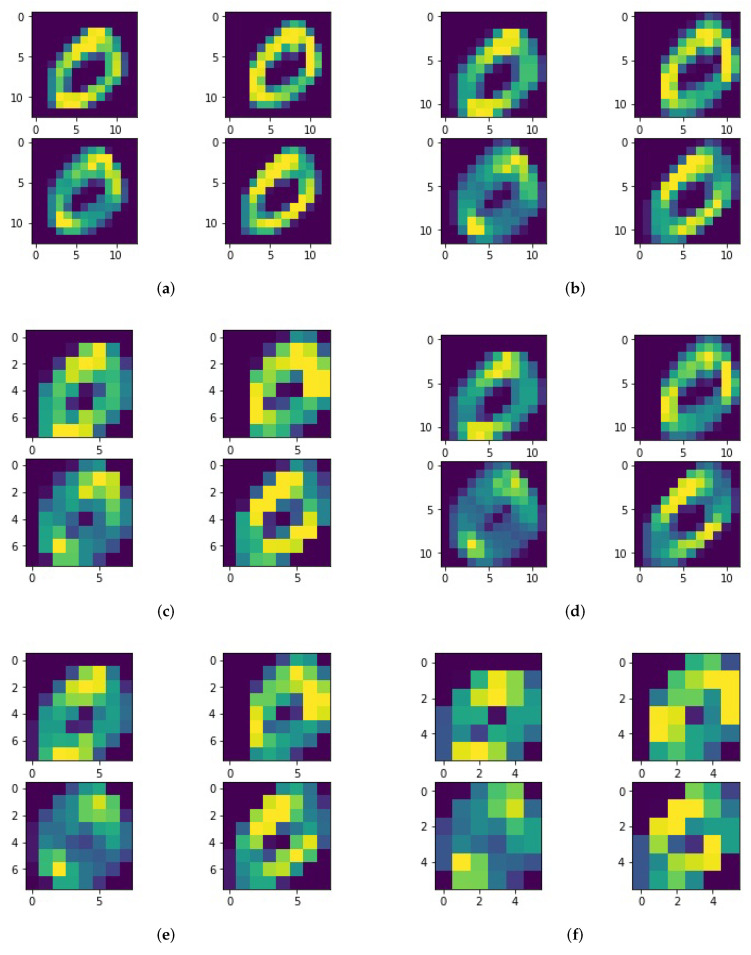
Effect of feature extraction after convolution and pooling. (**a**) 3×3 convolution kernel and 2×2 pooling; (**b**) 4×4 convolution kernel and 2×2 pooling; (**c**) 4×4 convolution kernel and 3×3 pooling; (**d**) 5×5 convolution kernel and 2×2 pooling; (**e**) 5×5 convolution kernel and 3×3 pooling; (**f**) 5×5 convolution kernel and 4×4 pooling.

Figure 7 shows the result of an image from the MNIST data-set which is labeled as a ‘0’ after the process of different configurations of the convolution layer and the pooling layer. The convolution layer includes four different convolution kernels (transverse, longitudinal and two cross diagonal weights distribution) of the same size, which is declared under each sub-figure. The moving step of the convolution kernel is 1. The size of the pooling layer is also declared under each sub-figure.

In order to find out the best configuration of convolution and pooling for our method, an experiment is conducted. Here we only consider the accuracy of a cluster trained using the bipolar supervised learning algorithm. The different configurations of the convolution and the pooling optimization phase are described below:

- Configuration 1: Convolution and pooling optimization is disabled;
- Configuration 2: Use 3×3 convolution kernel and 2×2 pooling;
- Configuration 3: Use 4×4 convolution kernel and 2×2 pooling;
- Configuration 4: Use 4×4 convolution kernel and 3×3 pooling;
- Configuration 5: Use 5×5 convolution kernel and 2×2 pooling;
- Configuration 6: Use 5×5 convolution kernel and 3×3 pooling;
- Configuration 7: Use 5×5 convolution kernel and 4×4 pooling.

Four different encoding methods have been designed to be used in the conversion layer of the Spiking Encoding Module of our SNN:

- Linear encoding method: $T_{spiking}=255-V$. Here use 255 to subtract the value of a pixel *V* in an image to get the spiking time for that pixel, because the image pixel values in the MNIST data-set range from (0, 255);
- Exponential encoding method: $T_{spiking}=\frac{255}{2^{V}}$;
- Inverse encoding method: $T_{spiking}=\frac{255}{V}$;
- Power encoding method: $T_{spiking}=\frac{255}{V^{2}}$.

**Figure 8:**
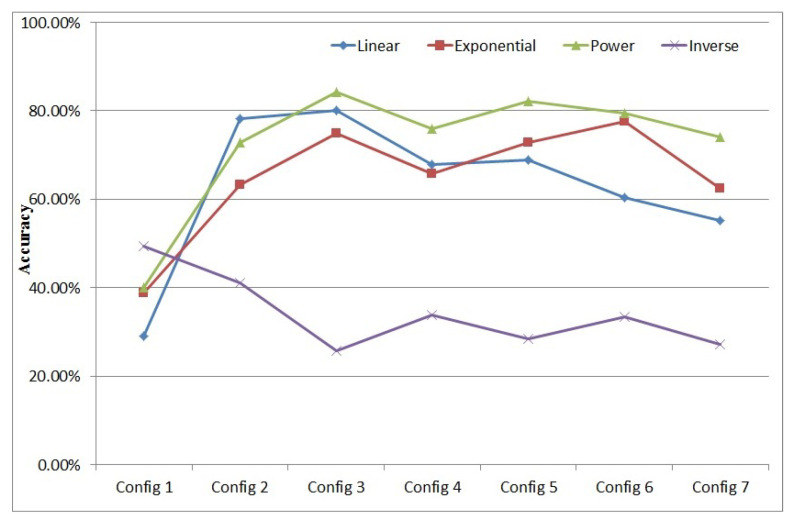
Efficiency of convolution and pooling optimization.

The comparison of accuracy for those seven configurations with different spiking encoding methods is shown in Figure 8. It can be seen from the results that, except for the inverse encoding method, if convolution and pool optimization is adopted, the accuracy of the results is much higher than that of the unadopted ones. Compared with other methods, the power encoding method has the best overall effect after using convolution and pooling optimization with different configurations.

Similarly, from the experimental results, we can see that under the configuration of convolution kernel size 4×4 and pooling size 2×2, the overall effect is better and the accuracy is higher than in other configurations.

According to its nature, the output of the inverse encoding method can easily be clustered in the first 20 ms. When no convolution or pooling optimization is involved (Configuration 1), the distribution of the feature information in the image is concentrated. Thus the inverse encoding method is more suitable and generates the best accuracy result. However, if convolution and pooling preprocessing is involved, information is more diversified, and more different image features are obtained, so the spiking time distribution is wider and not only focused on the first 20 ms, so the effect of the inverse function is reduced.

We therefore decided that the setting of the Spiking Encoding Module should be used with the configuration of convolution kernel size 4×4 and pooling size 2×2.

### 4.3. Evaluating the Timing of Encourage Teacher Signal and Punish Teacher Signal

In this paper, we proposed a reinforcement learning based bipolar supervised learning method for the synaptic learning of our SNN, where both encouraging teacher signals and punishing teacher signals would be used.

Thus, the timing of both the encouraging teacher signal and the punishing teacher signal needs to be carefully arranged. Both the encouraging teacher signal and the punishing teacher signal need to be added to neurons in layer 1 of the Spiking Decoding Module of each cluster.

In order to decide the optimal timing of both the encouraging teacher signal and the punishing teacher signal, an experiment is designed. Here we only consider the accuracy of a cluster trained using the bipolar supervised learning algorithm. For the setting of the Spiking Encoding Module, the convolution kernel size is 4×4, and the pooling size is 2×2.

**Figure 9:**
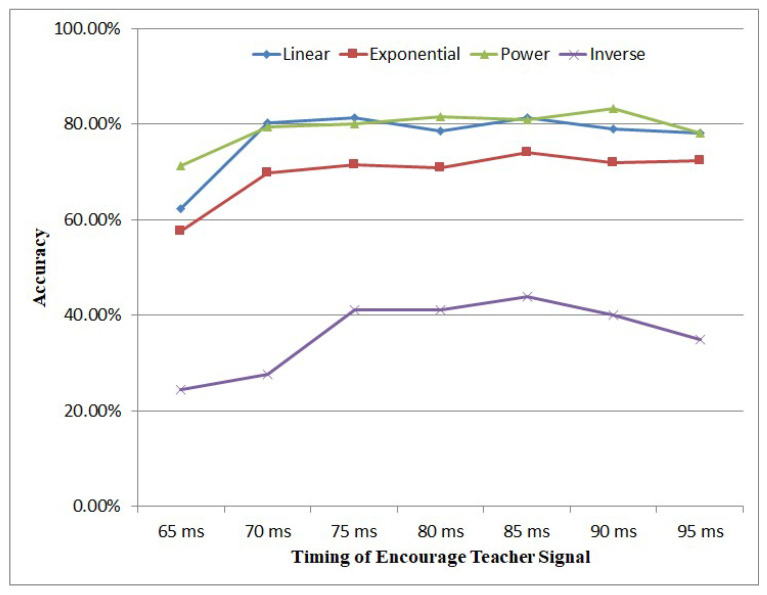
Influence of the timing of encouraging teacher signal.

Figure 9 shows the influence of the different timing of the encouraging teacher signal on accuracy. Since our spiking encoding principle is sending important information earlier, the spiking signals that represent the main features are concentrated in the front part of the time window. So it can be seen from the result in Figure 9 that, with the exception of the inverse encoding method, when the timing of encouraging teacher signal is arranged between (70 ms, 95 ms), the accuracy is not affected very much. Therefore, in this work, we set the timing of the encouraging teacher signal as 70 ms.

**Figure 10:**
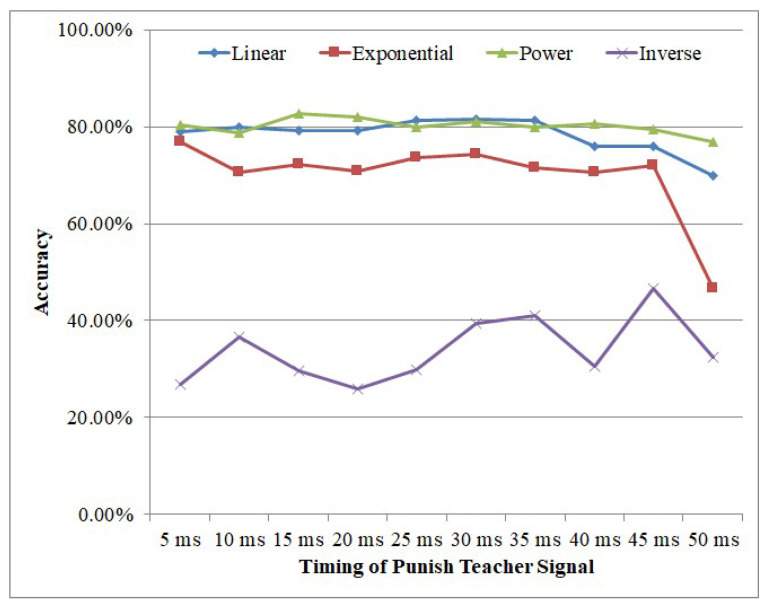
Influence of the timing of punishing teacher signal.

Figure 10 shows the influence of different timings of the punishing teacher signal on accuracy. It could be seen that, with the exception of the inverse encoding method, when the timing of the punishing teacher signal is arranged between (5 ms, 35 ms), the accuracy is not affected very much. Therefore, we set the timing of the punishing teacher signal at 35 ms in this work.

### 4.4. Evaluating the Number of Clusters in the SNN

As we discussed before, we explore the cluster-grouped structure mode to mimic the structural-and-functionalized tissue level of the human brain. More cluster might mean more powerful functionality, but this would consume more energy. We want to find a balance. We have conducted an experiment to decide the proper number of clusters in our SNN.

**Figure 11:**
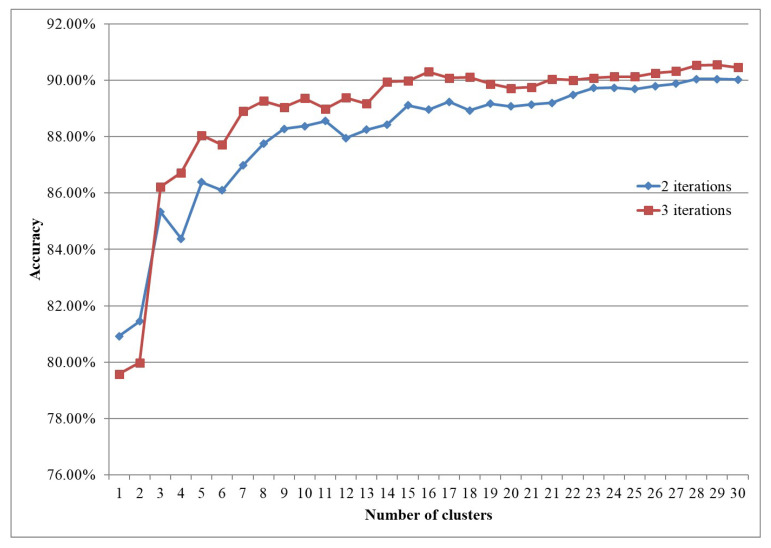
Influence of number of clusters.

Figure 11 shows the comparison for the different number of clusters.

Here the iteration refers to the training iteration for the construction of clusters. It can be seen that the results of the three iterations are better than the results of the two iterations, and the accuracy of the three iterations increases more quickly with the increase of the number of clusters.

Whether it is three iterations or two iterations, the accuracy can reach more than 90%. Also, when the number of clusters is more than 15, the improvement in accuracy is not too obvious, and the difficulty and complexity of network learning are increasing, so we determine that the number of clusters is 15.

### 4.5. Evaluating Threshold Value for Structural Learning

The biggest advantage of the biological neural network is that it can change its structure and connection according to the external environment, so it has huge flexibility and effectiveness. In this paper, we propose a structural learning process to achieve an adaptive SNN structure.

In order to determine the threshold value for structural learning, we have conducted a set of experiments. In this experiment, the number of clusters is set as 15. The power encoding method is used, and the setting of Spiking Encoding Module is using a convolution kernel size of 4×4 and a pooling size of 2×2.

**Figure 12:**
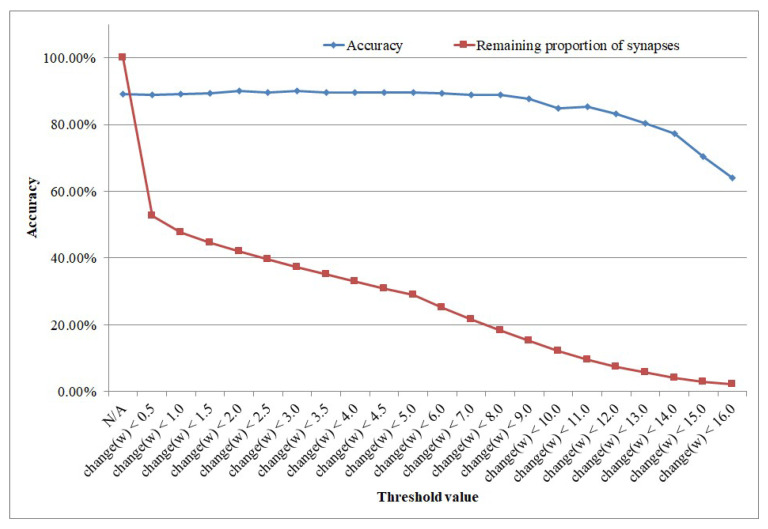
Influence of threshold value for structural learning.

Figure 12 shows the result. The vertical coordinates show the accuracy, while the transverse coordinates show different threshold configurations, where N/A represents the case where only synaptic learning is carried out.

It can be seen from the result that structural learning can not only improve the accuracy, but it also greatly reduces the complexity of the network in terms of the huge reduction in the number of synapses, as shown in Table 1. Only when the threshold of weight change has been set to larger than 10.0 would there be a gradual drop in accuracy. If we set the threshold of weight change as 9.0, then we could use a network of only 13,161 synapses to gain an accuracy of 88.80%, which is only slightly less than the accuracy of the network with 100% of the synapses connected, which is 86,400 synapses. Therefore, structural learning can largely enhance structural efficiency.

### 4.6. Comparison with Other Algorithms

In order to justify the ability of our SNN, several state-of-the-art SNN learning methods have been chosen for comparative purposes. The comparison with other methods is shown in Table 2. In order to maintain the consistence of the comparison, the calculation of neurons/synapses only considers the SNN part in each case.

According to the comparison, we could see that our method could generate reasonably high accuracy on the MNIST data-set with a small sample size and a small network scale. Ref. [26]’s work also involves a pruning process to enhance the learning ability and energy-efficiency of the SNN; however, our network structure is much smaller than theirs and has better accuracy. Ref. [27]’s accuracy is much better than ours, but their required training sample size is much larger than ours. Ref. [28] also used convolution and pooling layers to improve the feature extraction process, but we only used one layer of convolution and one layer of pooling; their process is much more complex. Ref. [29]’s work also generates better accuracy than ours, but they used a five-layer network (including four convolution layers), which is much more complex than ours. And their required sample size is much larger than ours as well.

## 5. Conclusions

In this paper, we have presented our method to build an adaptive SNN structure with small-sample learning ability. We have implemented a structural-and-synaptic co-learning method. The results show that we could generate better results when the required number of samples is only 1/4 of other methods. Furthermore, our SNN structure is more lightweight and thus more energy efficient.

In our method, since we use homogeneous clusters, it would be convenient to implement our SNN using neuromorphic hardware. The clusters would easily fit into the neuromorphic hardware array.

## Figures and Tables

**Table 1:** Effect of structural learning. N/A represents the case where only synaptic learning is carried out.

Threshold Value Setting	Accuracy	Remaining Proportion of Synapses	No. of Remaining Synapses
N/A	89.98%	100%	86,400
change(w) < 0.5	89.85%	52.62%	45,466
change(w) < 1.0	90.84%	47.54%	41,078
change(w) < 1.5	91.08%	44.23%	38,217
change(w) < 2.0	91.75%	42.53%	36,745
change(w) < 2.5	91.16%	39.32%	33,947
change(w) < 3.0	92.05%	36.76%	31,764
change(w) < 3.5	91.31%	35.23%	30,441
change(w) < 4.0	91.32%	33.76%	29,171
change(w) < 4.5	91.34%	30.82%	26,631
change(w) < 5.0	90.78%	28.13%	24,306
change(w) < 6.0	90.52%	25.14%	21,724
change(w) < 7.0	89.81%	21.23%	18,345
change(w) < 8.0	89.79%	17.98%	15,538
change(w) < 9.0	88.80%	15.23%	13,161
change(w) < 10.0	85.77%	12.65%	10,933
change(w) < 11.0	86.27%	9.32%	8055
change(w) < 12.0	84.06%	7.77%	6709
change(w) < 13.0	81.25%	5.21%	4504
change(w) < 14.0	78.14%	4.11%	3551
change(w) < 15.0	71.18%	2.98%	2573
change(w) < 16.0	64.85%	2.13%	1842

**Table 2:** Comparison results.

Algorithm	Unsupervised/Supervised	Required Sample Size	Layers	Neurons	Synapses	Accuracy
**Our Method**	Supervised	1000 × 15	2	586 × 15	31,764	92.05%
[20] (2013)	Supervised	60,000	Multiple	71,026	133,000,000	91.6%
[21] (2017)	Unsupervised	60,000	3	1584	314,000	84%
[23] (2017)	Unsupervised	60,000	3	1956	462,800	80.63%
[24] (2017)	Both	60,000	3	2394	1,270,400	89.7%
[25] (2019)	Supervised	60,000	2	2048	149,000	91.6%
[26] (2019)	Unsupervised	15,000	3	6400	192,000	91.5%
[27] (2019)	Unsupervised	270,000	4	1984	474,000	96.33%
[28] (2020)	Unsupervised	40,000	Multiple	800	160,000	92.2%
[29] (2021)	Supervised	60,000	5	15,238	161,996	98.74%

## Data Availability

Not applicable.
